# Treatment of Malarial Disease

**Published:** 1867-02

**Authors:** W. Anderson

**Affiliations:** Benjaminville, Ill.


*Treatment of Malarial Disease.*—By W. ANDERSON, M.D., of Benjaminville, Ill.

The commonly received opinion of medical writers and physicians generally, is, that the above class of diseases is of a zymotic character, or, at least, that they are produced by the continual presence, in the blood, of a morbific and foreign substance. Hence, medical men continually talk of cleansing the system by means of emetics, cathartics, cholagogues, diaphoretics, diuretics, etc.; and we hear such phrases as, "eradicating the disease," "driving it out of the system," and the like. The last article I read was devoted entirely to diuretics. It might do for old women and empirics to talk of such things, but certainly not for a philosophical physician. For convenience, I will confine myself to ague, as a type of the whole class. Some of the foregoing remedies, and others that might be mentioned, might serve a purpose in particular emergencies, but, in my humble opinion, they have little or no bearing on the disease itself, and are as often injurious, by weakening and otherwise injuring the system, as they are beneficial. Judging from experience and my opinion, (as well as that of some eminent writers,) antiperiodics are alone beneficial in all cases. Even cholagogues should be dispensed with, except the biliary symptoms are very prominent; for their secondary effect is to render the portal system more torpid, the same as cathartics exhaust the action of the bowels.

I do not derive these opinions from experience alone, but also from what I conceive to be the true nature of the disease. I do not believe malarious poison to be zymotic; that a small portion operates on the blood like contagion, and produces, catalytically, an indefinite quantity of a similar material, though this opinion is very generally received. Were it true, then, ague would be of the character of contagious diseases, to which it bears no possible resemblance, and, what is more certain, a very small portion of the morbific matter would produce the disease, and every individual in miasmatic districts would be troubled with the affection continually.

If this theory were true, it does not follow that depleting remedies are available, for who would think of eradicating variola or measles from the system in such a way, or of using them at all, except as palliating measures?

Nor is it probable that the removal of the cause will cure the disease. If a small portion of medicine breaks the paroxysms, by neutralizing the poison, it follows that a necessary quantity of the same medicine would neutralize all the poison in the system, and that there would be no relapse till farther exposure to the original cause, which we all know is not the fact. The morbific matter, if not continued by zymotic action or renewed by repeated exposure, probably does not remain in the system over thirty-six hours, yet, with the best treatment and in the purest atmosphere, the disease often lingers and is liable to return for many weeks. From this, it is reasonable to conclude that the commonly received opinion of medical men (and the learned Headland, in particular,) in regard to the action of quinia and other antiperiodics, namely, that they neutralize the malarial poison, etc., is not correct, and also that this disease is a nervous affection alone, which, when once established, is no longer dependent upon its original cause.

If these views are correct, it obviously follows that medicine which acts merely upon the blood, liver, etc., is of little or no avail in the ordinary treatment of the milder form of these disorders, and, when not available, are actually injurious, by weakening the system. These remarks, of course, do not apply to stimulants and tonics, many of which are antiperiodic in their nature and support the nervous system, which is doubtless the real and only seat of the disease, all other morbid phenomena being only symptoms.

A diversity of remedies may serve to amuse the patient, but in my practice I have but little use for anything save antiperiodics, with a very few palliatives during the paroxysms. Nor do I consider antiperiodics as specifics. I believe they merely support the nerves against a relapse till they regain their natural vigor. I believe they all act in the same manner, and that those which have sufficient power are about equally curative. Their order of efficiency is, in my opinion, as follows:—quinia, arsenic, salicine, etc. In all that has been written during the past twenty-five years, I think there has been no improvement on the plain, simple, and comprehensive plan of treatment, in ordinary agues and remittents, laid down by the illustrious Watson. His views of the nature of the disease, however, are not explicit, nor have they been much elucidated by more recent authors.

